# Polycomb subunit *Pcgf2* mediates ovulation and fertility through transcriptional regulation progesterone receptor

**DOI:** 10.3389/fcell.2022.1010601

**Published:** 2022-11-03

**Authors:** Yibo Wang, Wenji Wang, Kaixin Cheng, Kaiying Geng, Jing Liang, Peike Wang, Jiawei Zhang, Shudong Niu, Longzhong Jia, Shuo Zhang, Lingyu Li, Xiean Feng, Chao Wang, Haibin Wang, Hua Zhang, Yan Zhang

**Affiliations:** ^1^ State Key Laboratory of Agrobiotechnology, College of Biological Sciences, China Agricultural University, Beijing, China; ^2^ School of Life Science, Taizhou University, Taizhou, China; ^3^ Fujian Provincial Key Laboratory of Reproductive Health Research, Department of Obstetrics and Gynecology, The First Affiliated Hospital of Xiamen University, School of Medicine, Xiamen University, Xiamen, China

**Keywords:** PCGF2, granulosa cell, progesteron receptor, ovulation defect, subfertility

## Abstract

Ovarian follicles are the fundamental structure to support oocyte development, which provides mature oocytes for offspring. This process requires granulosa cells (GCs) to respond to the midcycle surge of hormones, leading to GC proliferation and differentiation by a series of genes’ transcriptional expression changes. Epigenetic mediator, Polycomb Repressive Complex 1 (PRC1) has been reported to function in fetal ovarian development. However, its functional relevance to folliculogenesis and ovulation remains unknown. In this study, we demonstrated that GC-selective depletion of PCGF2, a key component of PRC1, led to the loss of follicles, ovulation defects, and a lengthened estrus cycle, resulting in subfertility in female mice. The expression of PCGF2 is in the GCs of growing follicles and increases after human chorionic gonadotropin (hCG) stimulation. PCGF2 bound to the promoter of the key ovulation gene progesterone receptor (*Pgr*) and upregulated the expression of *Pgr* by targeting the epigenetic modification of H2AK119ub1 after hCG surge. Consistently, the expression of downstream genes of *Pgr* also sharply decreased, which resulted in the follicular rupture failed and oocyte entrapped in corpus luteum in GC-specific *Pcgf2* knockout mice. Together, our study identified that PCGF2 is essential for folliculogenesis and ovulation *via* modulating hormone receptor expression.

## Introduction

In mammalian ovaries, the ovarian follicle is the structural basis of female reproduction. It is composed of an oocyte and surrounded by granulosa cells (GCs) and theca cells (Eppig, 1991). The functions of GCs are crucial for folliculogenesis, including primordial follicles growing to preovulatory follicles, ovulation, and the luteal process under the effects of hormones (Zhang and Liu, 2015; Liu W. et al., 2017; Richards and Ascoli, 2018). GCs are also responsible for synthesizing and secreting hormones (Tureck and Strauss, 1982). The accurate control of specific gene transcriptional states is the basis for establishing a GC identity for folliculogenesis, ovulation, and corpus luteum formation (Park et al., 2004; Fan et al., 2009). The abnormal expression of genes of GC proliferation and differentiation results in serious ovarian dysfunction diseases such as polycystic ovary syndrome (PCOS) and premature ovarian insufficiency (POI) (Murdoch and Cavender, 1989; Wu et al., 2007; Schmidt et al., 2014; Duffy et al., 2019). However, the physiological significance of epigenetic regulators in GCs during follicular development has remained largely unexplored.

Polycomb group (PcG) proteins are chromatin-modifying proteins that act as critical epigenetic modulators (Entrevan et al., 2016; Piunti and Shilatifard, 2016). PcG proteins are classified into two multiprotein repressive complexes, namely polycomb repressive complexes 1 and 2 (PRC1 and PRC2) (Scheuermann et al., 2010; Aranda et al., 2015). In mammalian cells, PcG proteins mediate gene silencing through at least two distinct enzymatic activities directed to histone tails: PRC1 contains E3 ubiquitin ligase RING1A/B, which specifically targets histone H2A mono-ubiquitination at K119 (H2AK119ub1) (Cao et al., 2005; Taherbhoy et al., 2015), while PRC2 mediates histone H3 tri-methylation at K27 (H3K27me3) (Shen et al., 2008; Jiao and Liu, 2015).

The polycomb protein PCGF2 (polycomb group ring finger 2), also known as Mel-18, is a component of PRC1 and regulates stem cell differentiation and cancerous cell development (Morey et al., 2015). PCGF2 in luminal breast cancer cells positively regulates the promoter activities of ER-α (estrogen receptor-α) and PGR (progesterone receptor) by suppressing SUMOylation (Lee et al., 2015). Several recent studies reported that the PRC1 complex plays various roles in the female reproductive system (Baumann and De La Fuente, 2011; Yokobayashi et al., 2013; Tang et al., 2016). PCGF4 (polycomb group ring finger 4, also known as Bmi-1), a homolog of PCGF2, has redundant functional properties with PCGF2 (Liu X. et al., 2017). PCGF4 deficiency results in female infertility by activating p16/p19 signaling and increasing oxidative stress in mice (Wang et al., 2019). In female mice, PCGF4 interacts with PGR and E3 ligase, which is essential for normal embryo implantation independent of PRC1 repression function (Xin et al., 2018). Moreover, PRC1 regulates germ cell viability, meiosis onset, homologous chromosome synapsis, and sexual differentiation in different species (Baumann and De La Fuente, 2011; Yokobayashi et al., 2013; Tang et al., 2016; Maezawa et al., 2017). The findings suggest that PRC1 is a crucial epigenetic mediator for regulating hormone receptors during reproduction system development in female mice. However, it remains unclear whether the PRC1 complex is involved in regulating follicular development in the ovary.

In this study, we observed that *Pcgf2* is mainly expressed in ovarian GCs and then we established a GC-specific-*Pcgf2* knockout mouse model to investigate its physiological functions in the ovary *in vivo*. Our results showed that *Pcgf2* deficiency in granulosa cells results in subfertility, lengthened estrus cycle, and ovulation defects. In addition, we identified that *Pcgf2* regulates *Pgr* transcription in GCs under hCG surge, which is a key regulator in follicular rupture. Our study highlights that the mutation of *Pcgf2* may be a pathogenic factor of secondary premature ovarian insufficiency (POI) and anovulation.

## Methods and materials

### Animals

The C57BL/6J mice were acquired from the Laboratory Animal Center of the Institute of Genetics (Beijing, China). The *Foxl2-Cre* knock-in mice were obtained from the laboratory animal center, institute of zoology, Chinese Academy of Sciences. Detailed information of the generation of the *Foxl2-Cre* knock-in mouse was provided in Cen et al. (2020). The *Pcgf2* ^*fl/fl*^ mice were provided by Dr. Rongwen Xi (National Institute of Biological Sciences, Beijing). We crossed *Foxl2-Cre* with *Pcgf2* ^*fl/fl*^ to get *Foxl2-Cre;Pcgf2* ^*fl/fl*^ GC-specific *Pcgf2*-knockout mouse model. In *Pcgf2* ^*fl/fl*^ mice, *loxP-*flanked upstream the exon 5 expression in cells. When bred to *Foxl2-Cre* mice, the exon 5 of *Pcgf2* is deleted in the CRE-positive granulosa cells of all follicles. Mice were housed in mouse facilities that conformed to the standards and requirements of the Institutional Animal Care and Use Committee of China Agricultural University, No. AW92702202-3-1.

### Animal fertility testing

Mating trials were started at age of 6 months for female mice. Healthy males at 8–10 weeks of age were mated with females (1:2) for 7 months. The size and number of litter from each female were recorded to analyze the reproductive capacity.

### Evaluation of estrus cycle

The estrus cycle stage was evaluated every day by vaginal smear (Cora et al., 2015) for continuously 22 days. Using Wright’s stain, the stage was classified into 4 phases: Pro, proestrus; Est, estrus; Met, metestrus; Di, diestrus. The result of the estrus cycle was analyzed by using Graphpad prism 6.0 software.

### Ovulatory capacity testing

The female mice were superovulated by intraperitoneal injection of 5 IU pregnant mare serum gonadotropin (PMSG, Sansheng Biological Technology) at postnatal day 21 (PD21), and 46 h later the mice were additionally injected with 5 IU human chorionic gonadotropin (hCG, Sansheng Biological Technology). The oocytes were then collected from the oviducts, counted, and captured under the stereoscope. The number of oocytes was statistical analysis by using Graphpad prism 6.0 software. The ovaries were collected and analyzed the ovarian histology at PMSG 46 h, hCG 8 h, 12 h, 16 h, 24 h and 48 h.

### Histological analysis

Ovaries were collected at specific ages and then fixed in 4% Paraformaldehyde solution in PBS (PFA, sc-281692, Santa Cruz Biotechnology, United States). The embedded ovaries were dehydrated and then sectioned to obtain 8-µm thickness serial paraffin sections. The ovarian sections were processed to deparaffinize and rehydrate, the ovarian sections were stained with hematoxylin (sc-24973A, Santa Cruz Biotechnology, United States) to analyze the histology of the ovaries. The images were all analyzed under the microscope (DM500, Leica). Every five slices were counted for the number of primordial and primary follicles under 100 μm. Then, the counting numbers were multiplied by five to calculate the number of all primordial or primary follicles. Each slice was counted for the number of secondary follicles, antral follicles and the structure of the corpus luteum. The structures of the preovulatory follicle and oocyte-trapped CL were also counted in each slice. The number of follicles was statistically analyzed by using Graphpad prism 6.0 software.

### Meiotic resumption and cumulus expansion checking

The ovaries were collected at 8 h after human chorionic gonadotropin (hCG, Sansheng Biological Technology) injection when the meiotic resumption and cumulus expansion happened in most preovulatory follicles (Fan et al., 2009). The ovarian slices were stained with hematoxylin (sc-24973A, Santa Cruz Biotechnology, United States). To check the capacity of meiotic resumption, the number of germinal vesicle breakdown (GVBD) was counted in preovulatory follicles. The percent of GVBD was statistically analyzed by using Graphpad prism 6.0 software. In preovulatory follicles, the state of cumulus cell expansion was also checked at this time under the microscope (DM500, Leica).

### Immunofluorescence and immunohistochemistry staining

For immunofluorescence staining, the ovarian sections were subjected to deparaffinize and rehydrate. Using high temperature (95–98°C) in 0.01% sodium citrate buffer (pH 6.0) to retrieve the antigen of ovarian sections. Then, the ovarian sections were blocked by 10% donkey serum (Jackson ImmunoResearch) for 1 h and incubated with different primary antibodies at 4°C overnight. The primary antibodies used in this experiment were as follows: CX43 antibody (ab11370, rabbit, 1:300, Abcam, United Kingdom) and COLLAGEN IV antibody (ab6586, rabbit, 1:300, Abcam, United Kingdom), CYP11A1 antibody (Ab272494, rabbit, 1:300, Abcam, United Kingdom). Then the sections were incubated with Alexa Fluor 555- or 488-conjugated donkey secondary antibody (1:200, Life Technologies, United States) at 37°C for 60 min and then were counterstained with Hoechst 33342 (1:200, Beyotime, China). The images were photographed by using the Nikon Eclipse Ti digital fluorescence microscope or Andor Dragonfly spinning-disc confocal microscope and analyzed by software of Image J or Imaris.

For immunohistochemistry staining, we used the Rabbit two-step assay kit (PV-9001, ORIGENE, China). The ovarian sections were subjected to deparaffinize and rehydrate, and then retrieved the antigen. Then, the ovarian sections were incubated with endogenous peroxidase blocker for 10 min to block endogenous peroxidase. After washing with PBS for 9 min, the ovarian sections were then incubated with primary antibody H2AK119ub1 (D27C4, rabbit, 1:100, Cell signaling technology, United States) at 4°C overnight. Then the ovarian sections were incubated with reaction enhancing solution for 20 min at 37°C. After incubating goat anti-rabbit IgG polymer for 20 min at 37°C, we used the freshly prepared DAB color rendering solution to detect the H2AK119ub1 positive cells. The histological analysis was then checked after hematoxylin staining under the microscope (DM500, Leica).

### *In situ* hybridization

*In situ* hybridization was performed as described in Wang et al. (2006). For *in situ* hybridization, using Primer 5 software to design gene-specific primers using the following criteria if possible: the optimal fragment length is 200-300bp, the best annealing temperature is 60°C, and the GC content is 55%. The designed primers should preferably span exons, which can ensure that the mRNA is mature RNA and improve its specificity. We added T7 RNA polymerase promoter sequence 5′-AAT​TAA​CCC​TCA​CTA​AAG​GG-3′ to sense primer and added T3 RNA polymerase promoter sequence 5′-TAA​TAC​GAC​TCA​CTA​TAG​GG-3′ to antisense primer (See Supplementary Table S4) and order oligos. Using ovary cDNA sample to do standard PCR with primers for getting more than 400 ng product. Running PCR reaction out on a 1% agarose gel and cut out band corresponding to expected size amplicon. And then, we purified DNA from the gel using a gel extraction kit (TIANgel Purification Kit, Lot#W9812) and used nanodrop to quantify DNA concentration. The concentration of DNA should be higher than 20 ng/mL for doing the next step. Mouse-specific *Pcgf2* cRNA probe was labeled with digoxin by using DIG Northern Starter Kit (12039672910, Roche, Switzerland). To generate the sense probe of *Pcgf2*, we used T7 RNA polymerase in one reaction and used T3 RNA polymerase in a separate reaction to generate the antisense probe of *Pcgf2*. 10-μm ovarian cryosections hybridized with *Pcgf2* antisense probe to detect the *Pcgf2* mRNA localization and the sense probe served as negative control. After hybridization with cRNA probes for 24 h at 65°C, we used anti-digoxigenin-AP (75 mU/mL) to bind the DIG-label at 4°C overnight. After washing slices with MABT (maleic acid with 10%v/v Tween-20) solution for 1 h, we then incubated the slices with NBT/BCIP solution to react with the AP for color rendering, the positive signals were existed in purple in cytoplasm of the cells. To terminate the color rendering at a proper time, the slices were washed with PBS and then stained with Nuclear Fast Red solution for 10 min to display the nuclear of cells. And eventually the slices were dehydrated and transparent. The images were all analyzed under the microscope (DM500, Leica).

### Granulosa cells isolation

Granulosa cells were isolated by a follicular puncture from PMSG (5 IU, 46 h) and hCG (5 IU, 6 h and 16 h)-primed PD21 immature mice. Briefly, GCs at different times were released from antral follicles or corpus luteum by puncturing with a needle and the GCs were then stored at −80°C until being used.

### RNA isolation and quantitative real-time PCR

For quantitative real-time PCR (QRT-PCR), total RNA was isolated from GCs of at least 3 additional *GC-Pcgf2* ^*−/−*^ and *GC-Pcgf2* ^*+/+*^ female mice. Reverse transcription was done using the PrimeScript™RT reagent Kit with gDNA Eraser (TAKARA, PR047Q, Japan). The PCR reaction included 5 µL of SYBR Green Mix (Roche, 04913914001, Switzerland), cDNA product (1:10 dilution), and 0.5 µM of primers. Relative levels of mRNAs were calculated and normalized to the levels of endogenous β-actin in the same samples. The relative transcript levels of other samples were compared to the control, and the relative expression levels were shown in the graphs. For each experiment, QRT-PCR reactions were done in triplicate. The primers used in this paper were listed in Supplementary Table S4.

### Western blot

The GCs in different groups were collected and then lysed in WIP lysis solution (Cell signaling technology, United States). Separated the prepared protein samples by electrophoresis with 10% SDS-PAGE and transferred the protein to polyvinylidene fluoride (PVDF) membranes (IPVH00010, Millipore, United States). Then, blocked the membranes by using 5% nonfat-dry milk for 1 h at 37°C and incubated the membranes at 4°C overnight with the primary antibodies to be tested. The used primary antibody in the western blot was H2AK119ub1 (D27C4, rabbit, 1:1000, Cell signaling technology, United States). After overnight incubating, the membrane was washed with tris-buffered saline with 0.05% tween (TBST) for 30 min and then processed to incubate with the secondary antibody at room temperature for 1 h (1:5000, Beyotime, China). The level of α-Tubulin (A0208, rabbit, 1:1000, Beyotime, China) was used as an internal control. The membrane was washed with TBST for 30 min. The protein on membrane was captured by the Super Signal detection system (Prod 34080, ThermoFisher Scientific, United States). The images were then quantified by Image J software.

### Serum hormone assay

Mice were anesthetized and blood was collected by orbital blood. The level of progesterone (P4) in serum was analyzed by the Beijing Northern Institute of Biotechnology.

### ChIP-sequence analysis

ChIP-Sequence data were downloaded from Scelfo et al. (2019) (GEO: GSE122715) (Scelfo et al., 2019). Reads were aligned to the mouse reference genome mm9 for ChIP-Sequence of PCGF2 and H2AK119ub1 samples, using Bowtie2 v2.4.5 with default parameters without allowing for multi-mapping (–m 1). The PCR duplicates were removed by using PICARD (http://broadinstitute.github.io/picard/). The peaks were called by using MACS2 v2.2.7.1 with default parameters. A list containing the final PCGF2 and H2AK119ub1 peaks used in the analyses could be found in Supplementary Tables S1, S3. Genomic peak annotation was performed with the R package ChIPseeker v1.30.3, considering the region ± 2.0 kb around the TSS as the promoter. Gene ontology (Lydon et al.) analysis of PCGF2 targets was performed using the R package clusterProfiler setting as threshold an adjusted *p* value and q-value of 0.05. For visualization of PCGF2 enrichment of ChIP-seq, the BigWig file with input signal subtracted was generated using the function bamCompare from deepTools 3.5.1 with default parameters. Heatmap was performed using the functions computeMatrix followed by plotHeatmap from deepTools excluding blacklisted regions by ENCODE. Known motif discovery was performed by using HOMER v4.11 with default parameters.

## Results

### Granulosa cell-specific deletion of *Pcgf2* resulted in subfertility

**FIGURE 1:**
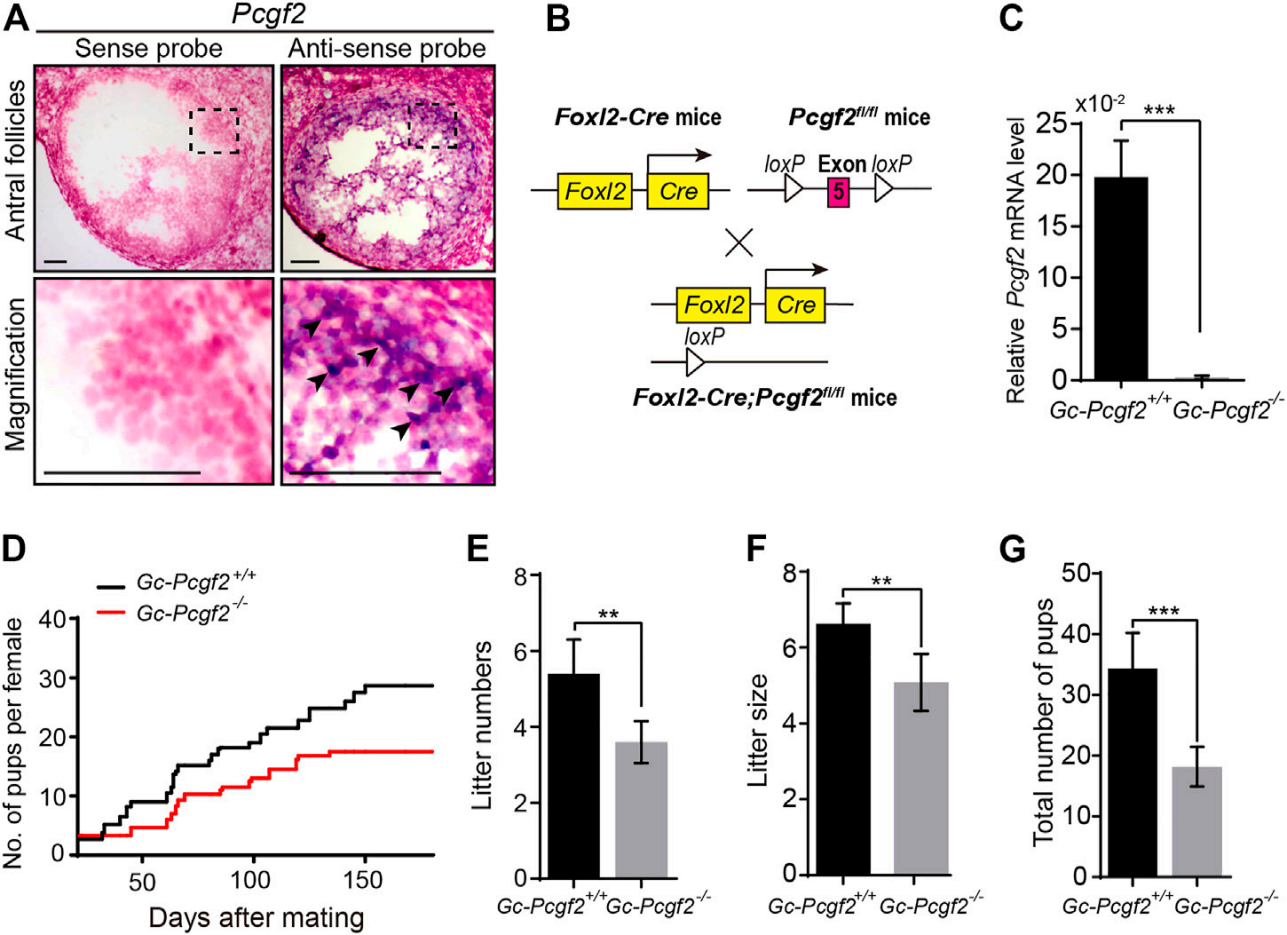
Deleting *Pcgf2* from ovarian follicle granulosa cells decreases fertility. **(A)** *In situ* hybridization showing *Pcgf2* mainly expressed in granulosa cells (arrowheads) of growing follicles (*n* = 3 per group). Scale bars: 50 µm. **(B)** Schematic diagram of deletion of *Pcgf2* exon 5 by *Foxl2-Cre*-mediated recombination in granulosa cells in *GC-Pcgf2* ^*−/−*^ ovaries. **(C)** Quantitative real-time PCR (QRT-PCR) result showing the mRNA level of *Pcgf2* sharply reduced in granulosa cells of *GC-Pcgf2* ^*−/−*^ ovaries (*n* = 3 per group). **(D–G)** Fertility checking showing a subfertility phenotype in the *GC-Pcgf2* ^*−/−*^ female mice after mating with healthy wild-type males at 6 months. **(D)** Fertility checking result showing a subfertility phenotype in the *GC-Pcgf2* ^*−/−*^ female mice (*n* = 5) with significantly decreased number and size of litters, compared with in *GC-Pcgf2* ^+/+^ mice (*n* = 5), during 7 months of mating. **(E)** Quantitative analysis result showing a significantly decreased number of litters in *GC-Pcgf2* ^*−/−*^ female mice (4 ± 0) compared with the controls (5 ± 1; *n* = 5 per group). **(F)** Quantitative analysis results showing reduced litter size in *GC-Pcgf2* ^*−/−*^ female mice (5 ± 1) compared with the controls (7 ± 0; *n* = 5 per group). **(G)** Quantitative analysis results showing a significantly decreased total number of pups in *GC-Pcgf2* ^*−/−*^ female mice (18 ± 3) compared with the controls (34 ± 5; *n* = 5 per group). **p < 0.01 and ***p < 0.001 by two-tailed unpaired Student’s *t* test.

To address the functional role of *Pcgf2* in folliculogenesis, we first analyzed the *Pcgf2* expression pattern in ovaries by *in situ* hybridization. *Pcgf2* was mainly detected in the GCs of growing follicles (Figure 1A and Supplementary Figure S1, arrowheads), suggesting as a potential role in folliculogenesis and fertility in females.

Next, we generated a GC-specific *Pcgf2* knockout mouse model, to explore the role of *Pcgf2* in ovaries. By flanking the exon 5 of *Pcgf2* with *loxP* sequences (*Pcgf2* ^*flox/flox*^) crossed with *Foxl2-Cre* mice, as illustrated in Figure 1B, we attained the resulting *Foxl2-Cre;Pcgf2* ^*fl/fl*^ mice, referred to as *GC-Pcgf2* ^*−/−*^. The littermates of *No-Cre;Pcgf2* ^*fl/fl*^ mice, referred to as *GC-Pcgf2* ^+/+^, were used as controls. Quantitative real-time PCR (QRT-PCR) was performed to detect the mRNA level of *Pcgf2* in GCs to validate the efficiency of deletion in *GC-Pcgf2* ^*−/−*^ female mice. QRT-PCR analysis showed that *Pcgf2* was effectively deleted in the GCs of ovarian follicles in *GC-Pcgf2* ^*−/−*^ mice (Figure 1C). These data suggested that the *GC-Pcgf2* ^*−/−*^ mouse model could be used to detect the functional role of *Pcgf2* in ovarian GCs. We tested female fertility by mating *GC-Pcgf2* ^*−/−*^ and *GC-Pcgf2* ^*+/+*^ female mice with healthy wild-type males. *GC-Pcgf2* ^*−/−*^ female mice showed a subfertility phenotype from the age of 6 months (Figure 1D). Compared with *GC-Pcgf2* ^*+/+*^ female mice, we found lower litter numbers (4 ± 0 in *GC-Pcgf2* ^*−/−*^ vs. 5 ± 1 in *GC-Pcgf2* ^*+/+*^; Figure 1E) and reduced litter sizes (5 ± 1 in *GC-Pcgf2* ^*−/−*^ vs. 7 ± 0 in *GC-Pcgf2* ^*+/+*^; Figure 1F) produced by *GC-Pcgf2* ^*−/−*^ female mice. These resulted in a decreased total number of pups in *GC-Pcgf2* ^*−/−*^ female mice (18 ± 3) compared with *GC-Pcgf2* ^*+/+*^ female mice (34 ± 5) after 7 months of mating trial tracing (Figure 1G). These results indicate that the *Pcgf2* function in GCs is involved in regulating female reproductive capacity.

### Deletion of *Pcgf2* from granulosa cells led to loss of follicles, ovulation defect, and a lengthened estrus cycle

**FIGURE 2:**
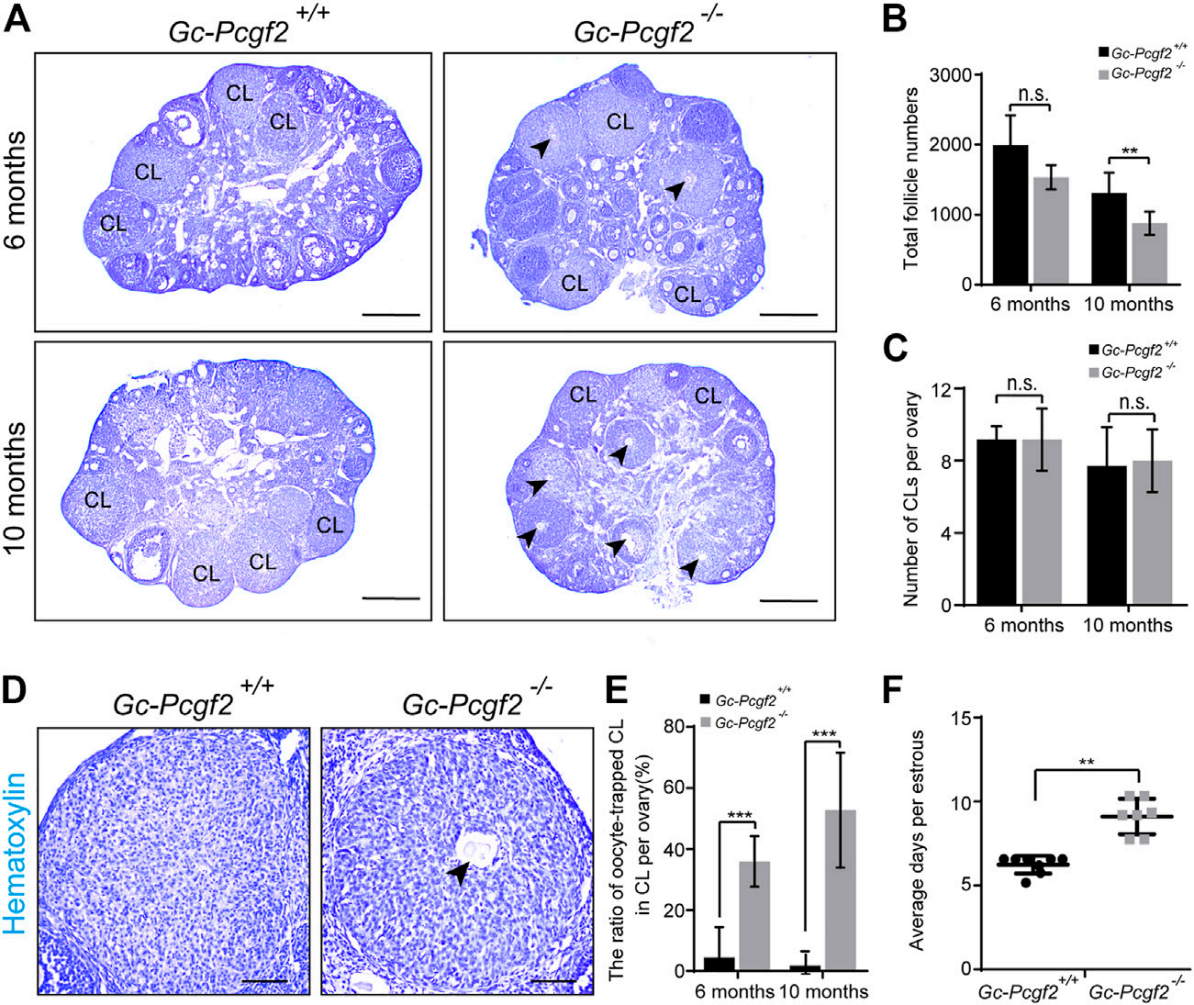
Deleting *Pcgf2* from ovarian follicle granulosa cells accelerates follicle loss and disrupts the estrus cycle. **(A)** Histological analysis showing the corpus luteum contained entrapped oocytes (arrowheads) were found in *GC-Pcgf2* ^*−/−*^ ovaries at 6 months and 10 months. CL, corpus luteum. Arrowheads, oocytes entrapped in CLs. Scale bars: 500 µm. **(B)** Follicle counting result showing identical numbers of the total follicle in female mice at 6 months (1585 ± 182 in *GC-Pcgf2* ^*−/−*^ vs. 1994 ± 379 in *GC-Pcgf2* ^*+/+*^; *n* = 5 per group) and a significantly decreased total follicle number in *GC-Pcgf2* ^*−/−*^ mice at 10 months (878 ± 156 in *GC-Pcgf2* ^*−/−*^ vs. 1310 ± 270 in *GC-Pcgf2* ^*+/+*^; *n* = 8 per group). **(C–E)** Histological analysis of corpus luteum showing significantly higher ratio of oocytes entrapped in CLs of *GC-Pcgf2* ^*−/−*^ ovaries, compared with those in the controls at 6 months and 10 months. **(C)** CL counting results showing an identical number of CLs in ovaries at 6 months (9 ± 2 in *GC-Pcgf2* ^*−/−*^ vs. 9 ± 1 in *GC-Pcgf2* ^*+/+*^; *n* = 6 per group) and 10 months (9 ± 2 in *GC-Pcgf2* ^*−/−*^ vs. 7 ± 2 in *GC-Pcgf2* ^*+/+*^; *n* = 8 per group). **(D)** Detailed histological analysis of CLs showing the structures of CL entrapped with oocyte (arrowheads) of *GC-Pcgf2* ^*−/−*^ ovaries at 6 months. Scale bars: 100 µm. **(E)** Quantitative result showing significantly higher ratio of oocyte-trapped-CL in *GC-Pcgf2* ^*−/−*^ (6 months: 36% ± 7%; 10 months: 53% ± 17%) compared with in the controls (6 months: 4% ± 9%; 10 months: 2% ± 4%; 6 months: *n* = 6 per group, 10 months: *n* = 8 per group). **(F)** Quantitative result showing a longer estrus cycle in *GC-Pcgf2* ^*−/−*^ (9 ± 1 day; *n* = 7) compared with in the controls (6 ± 0 days; *n* = 8). The experiments were repeated at least three times. The data in **(B,C,E,F)** represent the results (mean ± SD) of the biological triplicate experiments. n.s. *p* > 0.05, **p < 0.01, and ***p < 0.001 by two-tailed unpaired Student’s *t* test.

To find out the cause for subfertility in *GC-Pcgf2* ^*−/−*^ female mice, we detected the development of follicles in *GC-Pcgf2* ^*−/−*^ ovaries at different ages. A similar morphology of ovaries (Supplementary Figure S2A, PD6) and an identical total number of follicles were found in *GC-Pcgf2* ^*−/−*^ (3925 ± 812) and *GC-Pcgf2* ^*+/+*^(3748 ± 471) mice at postnatal day 6 (PD6) (Supplementary Figure S2B), suggesting that deletion of *Pcgf2* in (pre)GCs did not affect reproductive reserves formation. In PD35 and 2-month female mice, both ovarian size (Supplementary Figure S2A, PD35 and 2 months) and total numbers of follicles in *GC-Pcgf2* ^*−/−*^ were similar to those in the controls (Supplementary Figure S2B). However, follicle counting showed that the number of primordial follicles (PFs) was significantly reduced in *GC-Pcgf2* ^*−/−*^ ovaries (751 ± 126) compared with those in the controls (1181 ± 271) at 6 months (Supplementary Figure S2C). In 10-month female mice, fewer PFs and GFs distribution were found in *GC-Pcgf2* ^*−/−*^ ovaries (Figure 2A and Supplementary Figures S2B,C; PFs: 460 ± 115 in *GC-Pcgf2* ^*−/−*^ vs. 719 ± 171 in *GC-Pcgf2* ^*+/+*^; GFs: 418 ± 84 in *GC-Pcgf2* ^*−/−*^ vs. 591 ± 130 in *GC-Pcgf2* ^*+/+*^), which resulted in sharply decreased total follicle numbers in *GC-Pcgf2* ^*−/−*^ mice (878 ± 156 in *GC-Pcgf2* ^*−/−*^ vs. 1310 ± 270 in *GC-Pcgf2* ^*+/+*^) at 10 months (Figure 2B). These results indicated that *Pcgf2* deletion in GCs lead to accelerate follicle loss in female mice. However, histological analysis showed identical numbers of corpus luteum were formed in the ovaries of *GC-Pcgf2* ^*−/−*^ and *GC-Pcgf2* ^*+/+*^ female mice at 6 and 10 months (Figures 2A,C; 6 months: 9 ± 2 in *GC-Pcgf2* ^*−/−*^ vs. 9 ± 1 in *GC-Pcgf2* ^*+/+*^; 10 months: 9 ± 2 in *GC-Pcgf2* ^*−/−*^ vs. 7 ± 2 in *GC-Pcgf2* ^*+/+*^). These results suggest that the decline of *GC-Pcgf2* ^*−/−*^ fertility is endowed with an undefined mechanism.

We checked the histology of CLs and found the abnormal entrapped oocytes were present inside CLs in *GC-Pcgf2* ^*−/−*^ ovaries at 6 months (36% ± 7% in *GC-Pcgf2* ^*−/−*^ vs. 4% ± 9% in *GC-Pcgf2* ^*+/+*^) (Figures 2A,D,E, arrowheads). In 10-month *GC-Pcgf2* ^*−/−*^ ovaries, CL structures entrapped with oocyte were found, compared with those in the controls, at 10 months (53% ± 17% in *GC-Pcgf2* ^*−/−*^ vs. 2% ± 4% in *GC-Pcgf2* ^*+/+*^) (Figures 2A,E, arrowheads). These results revealed ovulation disorders in *GC-Pcgf2* ^*−/−*^ female mice. Moreover, the relative mRNA level of *Pcgf2* was significantly increased at the estrus stage compared with that in other cycling stages (Supplementary Figure S2E). We further monitored the estrus cycle of *GC-Pcgf2* ^*−/−*^ and control female mice for 22 days. We found that *GC-Pcgf2* ^*−/−*^ mice exhibited a lengthened estrus cycle, compared with the control mice (9 ± 1 day in *GC-Pcgf2* ^*−/−*^ vs. 6 ± 0 days in *GC-Pcgf2* ^*+/+*^) (Figure 2F), which lead to a lower number of litters. These results suggest that *Pcgf2* knockout in GCs results in defects of the ovulation and estrus cycle, thus leading to subfertility in *GC-Pcgf2* ^*−/−*^ female mice.

### Follicular rupture failed in *GC-Pcgf2* ^*−/−*^ females after human chorionic gonadotropin stimulation

**FIGURE 3:**
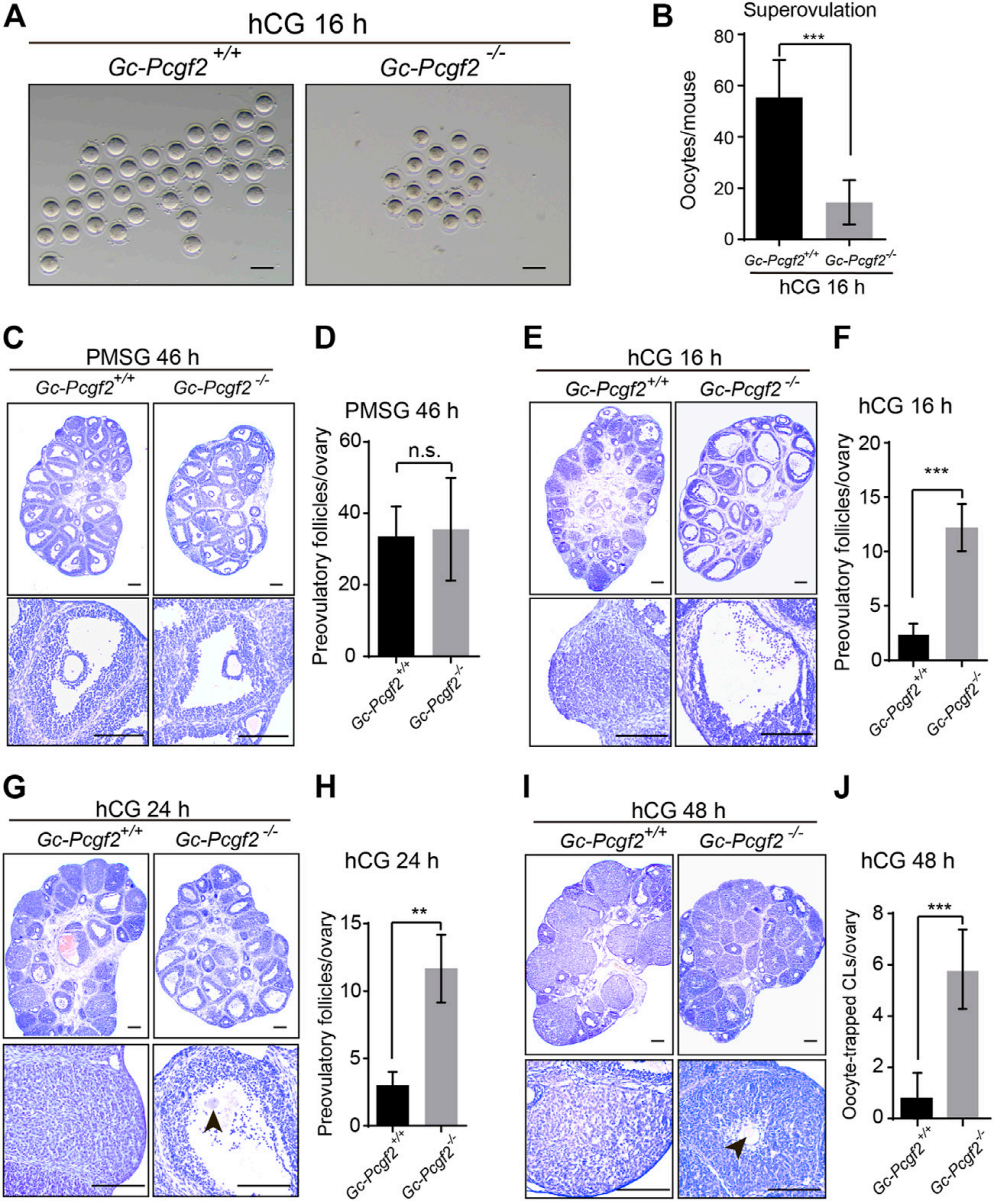
Abnormal response to hCG in granulosa cells of *GC-Pcgf2* ^*−/−*^ female mice. **(A)** Images showing ovulated oocytes from *GC-Pcgf2* ^*+/+*^ and *GC-Pcgf2* ^*−/−*^ female mice after superovulation. Scale bars: 100 µm. **(B)** Quantitative analysis results showing a decreased number of oocytes ovulated in *GC-Pcgf2* ^*−/−*^ female mice (16 ± 8) compared with in the controls (55 ± 14; *n* = 9 per group). **(C)** Histological analysis showing normal preovulatory follicles distributed in the ovaries of *GC-Pcgf2* ^*−/−*^ female mice at 46 h after PMSG (PMSG 46 h) treatment (*n* = 5 per group). Scale bars: 200 µm. **(D)** Follicle counting result showing an identical number of preovulatory follicles in *GC-Pcgf2* ^*−/−*^ (36 ± 13) and *GC-Pcgf2* ^*+/+*^ (34 ± 7) female mice at PMSG 46 h (*n* = 5 per group). **(E)** Histological analysis showing more preovulatory follicles in *GC-Pcgf2* ^*−/−*^ ovaries at hCG 16 h compared with those in the controls (*n* = 8 per group). Scale bars: 200 µm. **(F)** Statistical analysis result showing a significantly increased number of preovulatory follicles in ovaries in *GC-Pcgf2* ^*−/−*^ (12 ± 2) female mice, compared with those in the controls (2 ± 1) at hCG 16 h (*n* = 8 per group). **(G)** Histological analysis showing more preovulatory follicles in *GC-Pcgf2* ^*−/−*^ at hCG 24 h compared to those in the controls (*n* = 3 per group). Scale bars: 200 µm. **(H)** Quantitative analysis results showing a significantly increased number of preovulatory follicles in *GC-Pcgf2* ^*−/−*^ (12 ± 2) female mice, compared with those in the controls (3 ± 1) at hCG 24 h (*n* = 3 per group). **(I)** Histological analysis showing oocyte-trapped-CL (arrow) in ovaries in *GC-Pcgf2* ^*−/−*^ female mice at hCG 48 h compared with those in the controls (*n* = 7 per group). Scale bars: 200 µm. **(J)** Quantitative analysis results showing a significantly increased number of oocyte-trapped CL in *GC-Pcgf2* ^*−/−*^ (6 ± 1) ovaries at hCG 48 h compared with those in the controls (1 ± 1; *n* = 7 per group). The experiments were repeated at least three times. The data in **(B,D,F,H,J)** represent the results (mean ± SD) of the biological triplicate experiments. n.s. *p* > 0.05, **p < 0.01, ***p < 0.001 by two-tailed unpaired Student’s *t* test.

To examine the ovulatory capacity in *GC-Pcgf2* ^*−/−*^mice, we established the superovulation mouse model by injecting pregnant mare serum gonadotropin (PMSG) and human chorionic gonadotropin (hCG) at PD21. Statistical analysis of ovulated oocytes from oviducts showed that *GC-Pcgf2* ^*−/−*^ females only ovulated 16 ± 8 oocytes, which was significantly lower than that in *GC-Pcgf2* ^*+/+*^ mice (55 ± 14 oocytes) at 16 h after hCG treatment (hCG 16 h) (Figures 3A,B).

The ovarian histological observation showed that the number of preovulatory follicles in *GC-Pcgf2* ^*−/−*^ ovaries (36 ± 13) were no significance with the controls (34 ± 7) at 46 h after PMSG treatment (PMSG 46 h) at PD21 (Figures 3C,D). Next, hCG induced effective meiotic resumption (Supplementary Figures S3A,B, arrowheads) and cumulus expansion in *GC-Pcgf2* ^*−/−*^ ovaries (Supplementary Figure S3A) at 8 h after hCG treatment (hCG 8 h). Nonetheless, most of the preovulatory follicles failed to rupture in *GC-Pcgf2* ^*−/−*^ ovaries at 16 h after hCG treatment, compared with CL formation in *GC-Pcgf2* ^*+/+*^ ovaries (Figures 3E,F). We traced the fate of preovulatory follicles in *GC-Pcgf2* ^*−/−*^ ovaries and found that these follicles of *GC-Pcgf2* ^*−/−*^ did not release oocytes complex at 24 h after hCG injection (Figures 3G,H, arrowhead). Moreover, we counted the number of ovulated oocytes from oviducts at 24 h after hCG injection. Statistical analysis showed an identical number of ovulated oocytes at hCG 24 h compared to that at hCG 16 h in *GC-Pcgf2* ^*−/−*^ mice (Supplementary Figure S3C), indicating no more oocytes released after 16 h of hCG injection. Subsequently, we found oocyte-entrapped in the CLs (arrowheads) of *GC-Pcgf2* ^*−/−*^ ovaries at 48 h after hCG injection (Figures 3I,J; 6 ± 1 in *GC-Pcgf2* ^*−/−*^ vs. 1 ± 1 in *GC-Pcgf2* ^*+/+*^), which were similar to the structures of oocyte-trapped CLs in *GC-Pcgf2* ^*−/−*^ ovaries at 6 and 10 months. These data demonstrated that most of the preovulatory follicle fails to rupture in the *GC-Pcgf2* ^*−/−*^ ovaries of superovulation mouse models, even though the processes of meiotic resumption and cumulus expansion were normal.

### Loss of *Pcgf2* in granulosa cells affects granulosa cells loosen at ovulated side and luteinization process

**FIGURE 4:**
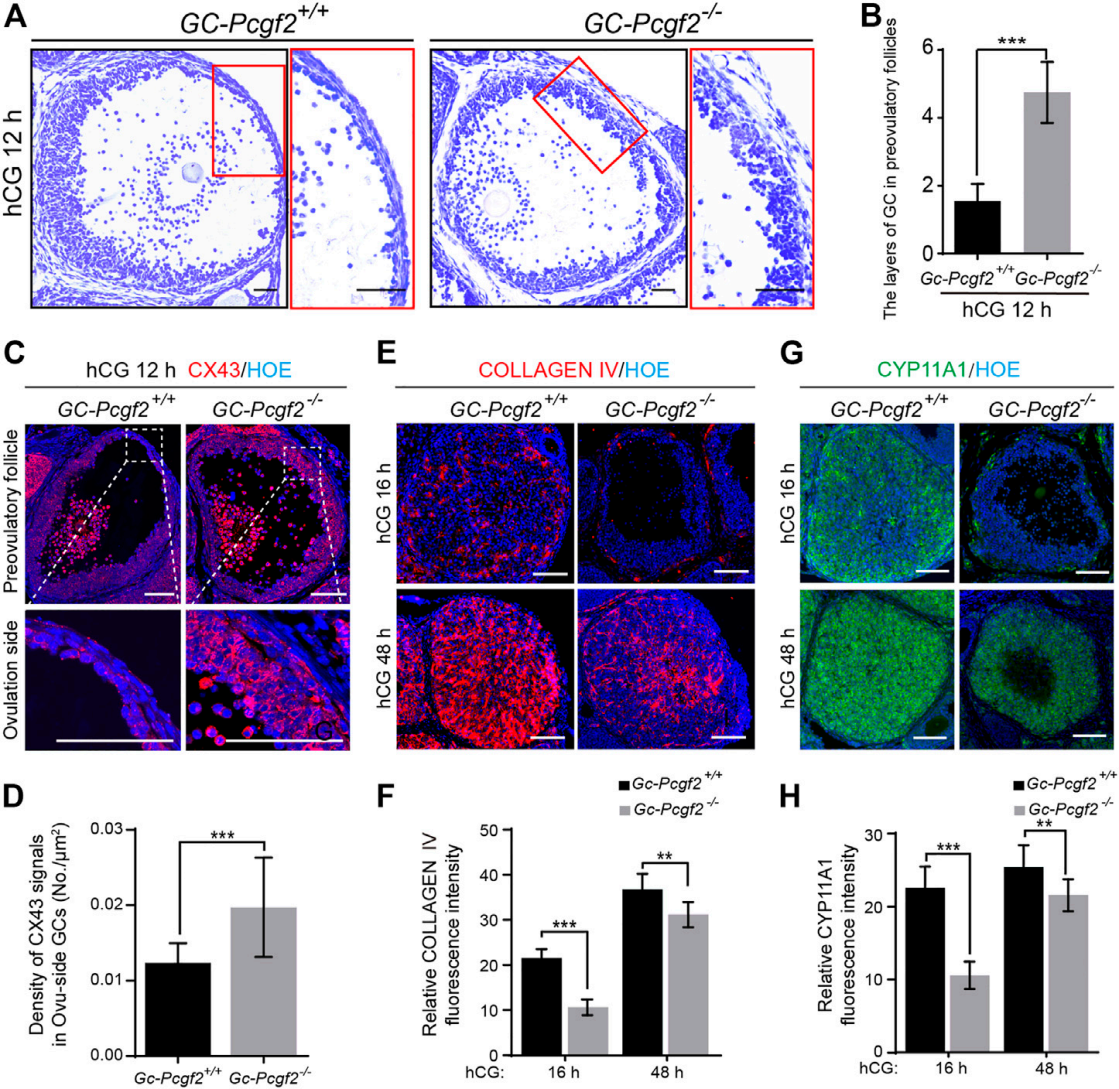
Deletion of *Pcgf2* in granulosa cells leading to follicular rupture fail and corpus luteum formation delay. **(A)** Histological analysis showing a thicker GC layer in ovulated-side (Ovu-side) of *GC-Pcgf2* ^*−/−*^ preovulatory follicles at hCG 12 h. Scale bars: 50 µm. **(B)** Statistical analysis result showing more layers of GC in Ovu-side of *GC-Pcgf2* ^*−/−*^ (5 ± 1) compared with that of the controls (2 ± 0; *n* = 40 per group). **(C)** Immunostaining showing decreased density of CX43 signals in Ovu-side GCs of *GC-Pcgf2* ^*−/−*^ compared with that of the controls. Red: CX43; Blue: Hoechst. Scale bars: 100 µm. **(D)** Quantitative analysis result showing the decreased density of CX43 signals in Ovu-side GCs of *GC-Pcgf2* ^*−/−*^ compared with that of the controls (*n* = 27 per group). **(E)** Immunostaining analysis result showing decreased expression of COLLAGEN IV in CLs of *GC-Pcgf2* ^*−/−*^ compared with that of the controls. Red: COLLAGEN IV; Blue: Hoechst. Scale bars: 100 µm. **(F)** Quantitative analysis result showing decreased fluorescence intensity of COLLAGEN IV in CLs of *GC-Pcgf2* ^*−/−*^ compared with that of the controls (hCG 16 h: *n* = 9 per group; hCG 48 h: *n* = 7 per group). **(G)** Immunostaining analysis result showing decreased expression of CYP11A1 in CLs of *GC-Pcgf2* ^*−/−*^ compared with that of the controls. Green: CYP11A1; Blue: Hoechst. Scale bars: 100 µm. **(H)** Quantitative analysis result showing decreased fluorescence intensity of CYP11A1 in CLs of *GC-Pcgf2* ^*−/−*^ compared with that of the controls (hCG 16 h: *n* = 10 per group; hCG 48 h: *n* = 12 per group). The data in **(B,D,F,H)** represent the results (mean ± SD) of the biological triplicate experiments. **p < 0.01, and ***p < 0.001 by two-tailed unpaired Student’s *t* test.

To investigate the mechanism of follicular rupture defects, we compared the morphology of GCs at the ovulated side (Ovu-side) of preovulatory follicles in *GC-Pcgf2* ^*−/−*^ and *GC-Pcgf2* ^*+/+*^ ovaries at 12 h after hCG stimulation, when they began to ovulate oocytes and form corpus haemorrhagicum (Sekulovski et al., 2020). We found that only 2 ± 0 layers of GCs remained at the Ovu-side of preovulatory follicles in *GC-Pcgf2* ^*+/+*^ovaries (Figures 4A,B, left). In contrast, 5 ± 1 layers of GCs existed at the Ovu-side of preovulatory follicles in *GC-Pcgf2* ^*−/−*^ ovaries (Figures 4A,B, right). This result showed that the loosening process of mural granulosa cells (mGCs) at the Ovu-side defected in *GC-Pcgf2* ^*−/−*^ female mice. We detected the distribution of gap junction protein connexin 43 (CX43) at the Ovu-side of preovulatory follicles at 12 h after hCG stimulation. In the preovulatory follicles of the controls, we observed almost no distribution of CX43 in the GCs of the Ovu-side (Figure 4C, left), indicating that the connection of GCs repressed before follicle ruptured. However, CX43 was highly expressed in the plasma membrane of GCs at the Ovu-side in *GC-Pcgf2* ^*−/−*^ mice compared with those in the controls (Figures 4C,D). These data suggested that GCs connect tightly with each other by gap junctions in *GC-Pcgf2* ^*−/−*^ ovaries, which results in the failure of follicular rupture.

Furthermore, we detected the subsequent luteinization process in *GC-Pcgf2* ^*−/−*^ and *GC-Pcgf2* ^*+/+*^ovaries. The immunofluorescent staining of CL markers, COLLAGEN IV (Figures 4E,F), and Cytochrome P450 family 11 subfamily A member 1 (CYP11A1, Figures 4G,H) showed a significant decline of fluorescence intensity in *GC-Pcgf2* ^*−/−*^ ovaries, compared with *GC-Pcgf2* ^*+/+*^ovaries at 16 h and 48 h after hCG treatment. These data showed that the luteinization process was delayed in *GC-Pcgf2* ^*−/−*^ ovaries. CYP11A1 is a member of the cytochrome P450 superfamily of enzymes that regulates the production of progesterone (P4), which is crucial for female fertility. Hormone detection showed significantly decreased concentrations of progesterone (P4) in *GC-Pcgf2* ^*−/−*^ female mice than in the controls both at 16 h and 48 h after hCG treatment (Supplementary Figure S4). These results indicate that *Pcgf2* in GCs is essential for follicular rupture and luteinization.

### *Pcgf2* transcriptional regulated progesterone receptor to mediate ovulation

**FIGURE 5:**
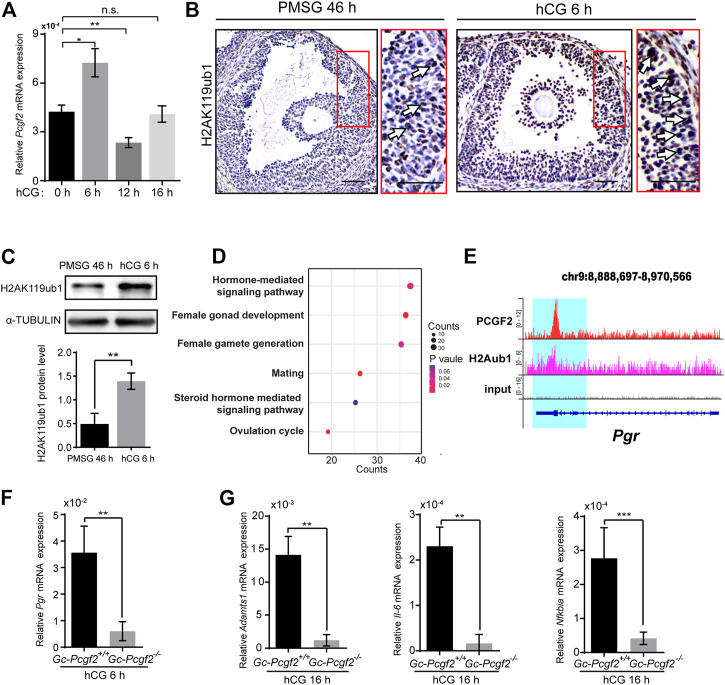
PCGF2 binding to the promoter of progesterone receptor and mediating the process of ovulation in mice. **(A)** QRT-PCR result showing a *Pcgf2* mRNA expression peak at hCG 6 h. **(B)** The immunohistochemical histological analysis of H2AK119ub1 showing an increasing signal of H2AK119ub1 (arrows) in the GCs of preovulatory follicles at hCG 6 h compared with at PMSG 46 h. Scale bars: 50 µm. **(C)** Quantitative analysis of western blot showing a significantly increasing accumulation level of H2AK119ub1 in GCs at hCG 6 h compared with at PMSG 46 h. **(D)** The GO analysis of PCGF2 target genes showing that the target genes were associated with female fertility, including ovulation and hormone related pathways. **(E)** Genomic snapshot of the indicated ChIP-seq profiles of PCGF2 and H2Aub1 (H2AK119ub1) at gene loci of *Pgr*. **(F)** The statistical analysis of the QRT-PCR result showing a sharply decreasing of *Pgr* in GCs of *GC-Pcgf2* ^*−/−*^ at hCG 6 h compared with that in controls (*n* = 3 per group). **(G)** The statistical analysis of QRT-PCR results showing significantly decreasing the mRNA levels of *Adamts1*, *Il-6*, and *Nfκbia* in GCs of *GC-Pcgf2* ^*−/−*^ at hCG 16 h. The experiments were repeated at least three times. The data in **(A,C,F,G)** represent the results (mean ± SD) of the biological triplicate experiments. n.s. *p* > 0.05, *p < 0.05, **p < 0.01, and ***p < 0.001 by two-tailed unpaired Student’s *t* test.

To uncover the molecular mechanism of *Pcgf2* mediating ovulation, we performed QRT-PCR to detect the mRNA expression dynamics of *Pcgf2* in GCs at different times after hCG injection. The highest mRNA level of *Pcgf2* in GCs was at 6 h after hCG injection (hCG 6 h) compared with hCG 0 h, 12 h, and 16 h (Figure 5A). This result implies *Pcgf2* mainly functions at 6 h after hCG stimulation during the ovulation process.

*Pcgf2* is required for stability of the PRC1 complex core which targeting at H2AK119ubl (Cao et al., 2005; Taherbhoy et al., 2015). Immunohistochemical staining showed significantly increased accumulation level of H2AK119ub1 in the GCs of preovulatory follicles at 6 h after hCG stimulation (hCG 6 h), compared with at 46 h after PMSG stimulation (PMSG 46 h) (Figure 5B, arrows). Western blot confirmed the sharply increased the level of H2AK119ub1 in GCs at hCG 6 h compared with PMSG 46 h (Figure 5C). These results imply that *Pcgf2* may function in ovulation by targeting H2AK119ub1 epigenetic modification at 6 h after hCG stimulation.

To find potential target genes associated with the ovulation pathways of PCGF2, we analyzed the publicly available ChIP-seq data of PCGF2 using the GEO database (GEO: GSE122715) (Scelfo et al., 2019). Visualization of PCGF2 enrichment by ChIP-seq analysis showed that PCGF2 had unique target loci around ± 2.0 kb of the TSS of common (Supplementary Figures S5A,B). PCGF2 had an extensive binding repertoire with 4341 target genes (Supplementary Table S1). We next enriched these PCGF2 target genes in a gene ontology analysis and selected female reproduction biology pathways to screen ovulation-related genes (Figure 5D and Supplementary Table S2). PCGF2 target genes were enriched with GO terms including “reproductive structure development”, “positive regulation of hormone secretion”, and “ovulation cycle” (Figure 5D). Among the enriched PCGF2 target genes of the ovulation cycle biology pathway, we found one of the key ovulation genes that were reported to be the main mediator of follicular rupture, progesterone receptor (*Pgr*), which expression was induced by luteinizing hormone (LH) (Park et al., 2020) (Supplementary Table S2). Park et al. (2020) reported the phenotypes of ovulation failed and the oocyte remains within CL in GC-specific *Pgr* knockout mice, which was similar to the phenotype of *GC-Pcgf2* ^*−/−*^ mice. The call peak result of PCGF2 detected a binding peak (Figure 5E) and a specific binding motif (Supplementary Figure S5C) at the promoter of *Pgr* (+457) (Supplementary Table S1). These results imply that PCGF2 regulates the expression of *Pgr* to affect ovulation.

We then collected GCs of preovulatory follicles and detected the mRNA level of *Pgr* at 6 h after hCG stimulation. The QRT-PCR result showed the mRNA level of *Pgr* in GCs of *GC-Pcgf2* ^*−/−*^ unexpectedly sharply decreased compared with that in control mice (Figure 5F), which was inconsistent with PRC1 mediated gene silencing as previous studies reported. This result suggested that PCGF2 upregulated the expression of *Pgr* during the ovulation process through nonclassical PRC1 functions. Furthermore, we found that the mRNA level of *Pgr* downstream genes, including *Adamts1* (Robker et al., 2000), *Il-6* and *Nfκbia* (Park et al., 2020) significantly reduced at 16 h after hCG treatment (Figure 5G). These data uncover that PCGF2 mediates ovulation by transcriptional regulation of key ovulation gene *Pgr* in mice.

## Discussion

Ovulation is a complex process with multiple cell lineage responding to luteinizing hormone (LH) surge, including the resumption of oocyte meiosis, the expansion of cumulus oocyte complex, preovulatory follicle rupture, and release of COCs. Many studies focus on the molecular mechanism of meiotic resumption and COC expansion, but little is known about the regulation mechanism of follicle rupture (Richards and Ascoli, 2018). Our study demonstrated that PCGF2 is highly expressed in GCs and affects follicle rupture through loosening GCs at the Ovu-side of preovulatory follicles. Ovulated cell behaviors are dependent on tissue remodeling hormone receptor interactions and specifically timed gene expression in distinct type cells. We provide physiological evidence that PCGF2 affects follicular rupture under LH surge *via* modulating *Pgr* transcriptional expression level.

The LH surge induces the high expression of key ovulation gene *Pgr* in the mGCs of preovulatory follicles (Park et al., 2020). As a nuclear receptor transcription factor, PGR regulates ovulation, the development of mammary glands, and embryo implantation through tissue-specific transcriptionally regulating downstream genes (Lydon et al., 1995; Lee et al., 2015; Xin et al., 2018). *Pgr* deletion in GCs does not affect the expansion of COC and the fertilization ability of oocytes. PGR regulates follicle rupture through induction of ovulation target genes, such as *Adamts1* (Doyle et al., 2004), *Edn2* (Ko et al., 2006), *IL-6* (Park et al., 2020), and *Pparg* (Kim et al., 2008). These are consistent with the phenotype of *GC-Pcgf2* ^*−/−*^ female mice. However, the transcriptional regulation mechanisms of *Pgr* expression in GCs induced by the LH surge are still not clear. We found that PCGF2 transcriptionally upregulates the expression of *Pgr* after hCG stimulation, rather than the classic function of PRC1 complex repressed gene expression. Moreover, the activation of *Pgr* promoter needs the interactions of the obligatory Sp1/Sp3 loci between the proximal promoter and the distal promoter by LH surge induction (Sriraman et al., 2003). Our ChIP analysis showed that PCGF2 and H2AK119ub1 enriched at the promoter region of PGR (+361/+437) between the proximal promoter and the distal promoter (Figure 5E, Supplementary Table S3). These data imply that the PCGF2 serve as a coregulator with LH mediated the transcription of *Pgr* in GCs by promoting Sp1/Sp3 interactions. However, *Pgr* gene expression was not completely suppressed in *GC-Pcgf2* ^*−/−*^ ovaries, perhaps because of the redundant functional properties of PCGF4 with PCGF2.

The ChIP-seq data showed that PCGF2 extensively binds to genes with accumulation of H2AK119ub1, suggesting that PCGF2 is involved in multiple functions of follicle development. We found that the reproductive capacity and follicle reserve of *GC-Pcgf2* ^*−/−*^ female mice decreased after 6 months compared to the controls. These results suggested that *GC-Pcgf2* ^*−/−*^ ovaries exhibited some symptoms of secondary POI after the age of 6 months. Previous studies discovered that the expression of aging-associated of genes such as *Egfr*, *Egr2*, *Tmed2*, and *Ptgs2* in GCs of aged primate ovaries were increased compared with young GCs (Wang et al., 2020). Our ChIP-seq data supports that these genes to be target of PCGF2 (Supplementary Table S1). This result implies that PCGF2 represses silencing of these aging-associated genes’ expression in GCs for maintaining the reproductive capacity of ovaries.

In summary, our study provides systematic *in vivo* evidence that polycomb subunit *Pcgf2* mediates ovulation and fertility through transcriptionally regulation progesterone receptor expression in female mice. Deletion of *Pcgf2* in GCs affects the follicular rupture and leads to a series of defects in female fertility. These results remind us that the mutation of *Pcgf2* may be a pathogenic factor of anovulation, such as luteinized unruptured follicle (LUF) syndrome. This study provides a potential target for finding and curing patients with anovulation in clinical treatment.

## Data Availability

The datasets presented in this study can be found in online repositories. The names of the repository/repositories and accession number(s) can be found in the article/Supplementary Material.
