# Supplementary material for: Polycomb subunit Pcgf2 mediates ovulation and fertility through transcriptional regulation progesterone receptor
Source: Front Cell Dev Biol. 2022 Nov 3;10:1010601. doi: 10.3389/fcell.2022.1010601 (PMC9669581; doi:10.3389/fcell.2022.1010601)
Supplement: Supplementary file 1 [file DataSheet2.pdf]

Supplementary Figure S1.

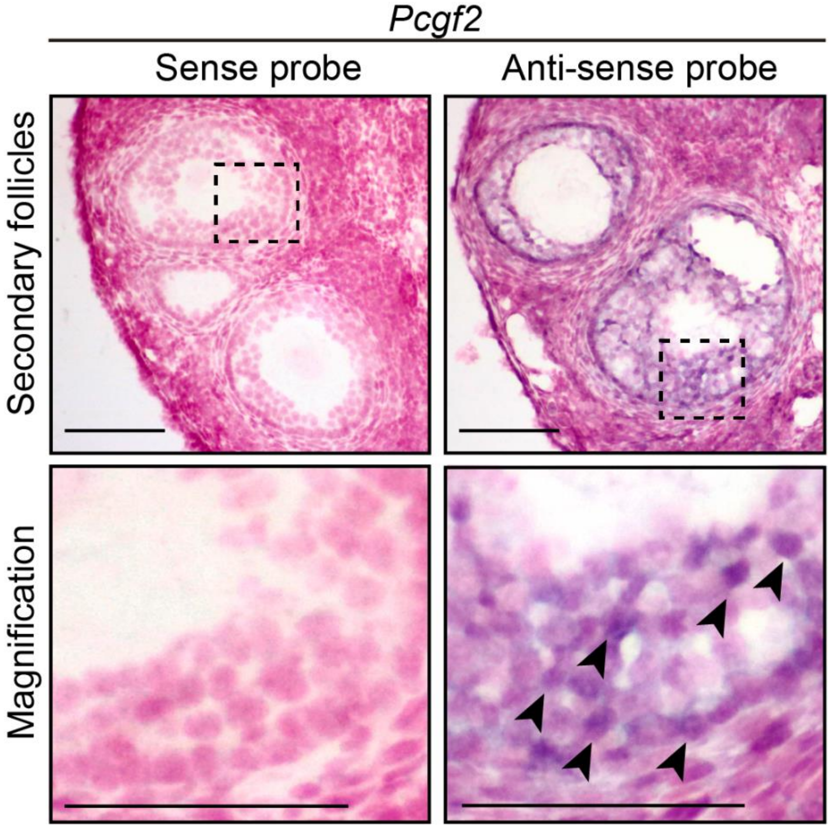

Supplementary Figure S1. *In situ* hybridization of *Pcgf2* in mouse ovary. *In situ* hybridization results showing *Pcgf2* mainly expressed in granulosa cells (arrowheads) of growing follicles (n = 3 per group). Red: nucleus, purple: *Pcgf2* positive signals. Scale bars: 50 μm.

Supplementary Figure S2.

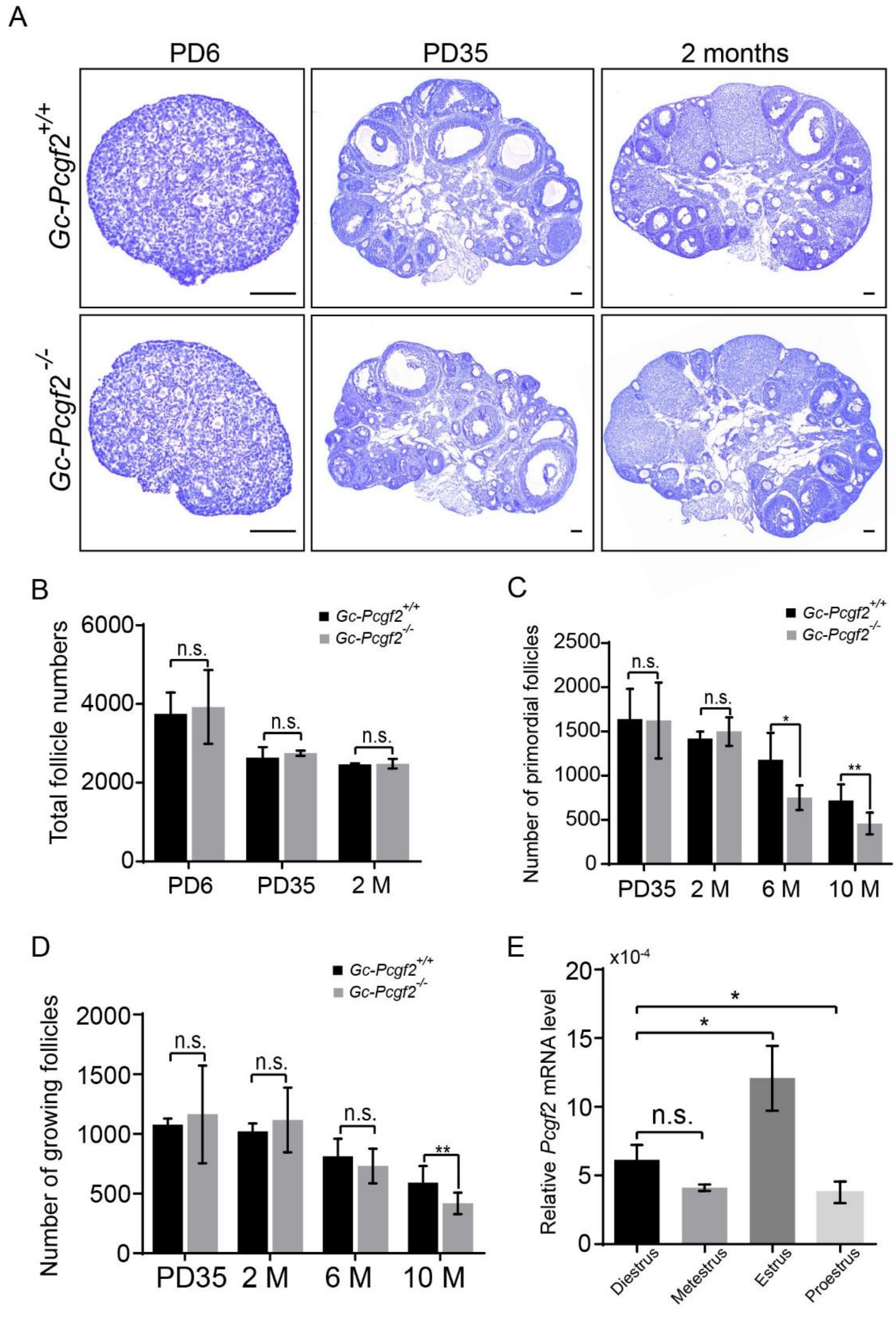

Supplementary Figure S2. The number of follicles decreases sharply in aged *GC-Pcgf2*$^{-/-}$ mice. (**A**) Histological analysis of ovaries showing normal follicle distribution in ovaries at PD6, PD35, and 2 months in *GC-Pcgf2*$^{+/+}$ and *GC-Pcgf2*$^{-/-}$ mice. Scale bars: 100 µm. (**B**) Follicle counting results showing an identical number of total follicles in ovaries of *GC-Pcgf2*$^{-/-}$ female mice at PD6, PD35 and 2 months, compared with the controls (n = 3 per group). (**C**) Follicle counting results showing an identical number of primordial follicles in ovaries of *GC-Pcgf2*$^{-/-}$ female mice, compared with

the controls, at PD35 and 2 months (n = 3 per group). In 6-month and 10-month *GC-Pcgf2*$^{-/-}$ female mice, the number of primordial follicles significantly decreased compared with the controls (6-month: n = 5 per group; 10-month: n = 8 per group). (**D**) Follicle counting results showing identical numbers of growing follicles in ovaries of *GC-Pcgf2*$^{-/-}$ female mice, compared with the controls, at PD35 (n = 3 per group), 2 months (n = 3 per group) and 6 months (n = 5 per group). The number of growing follicles significantly decreased at 10 months in ovaries of *GC-Pcgf2*$^{-/-}$ female mice compared with the controls (n = 8 per group). (**E**) QRT-PCR result showing the *Pcgf2* mRNA expression in granulosa cells during estrus cycles. The experiments were repeated at least three times. The data represent the results (mean ± SD) of the biological triplicate experiments. n.s. $P > 0.05$, *$P < 0.05$, **$P < 0.01$ by two-tailed unpaired Student's *t* test.

Supplementary Figure S3.

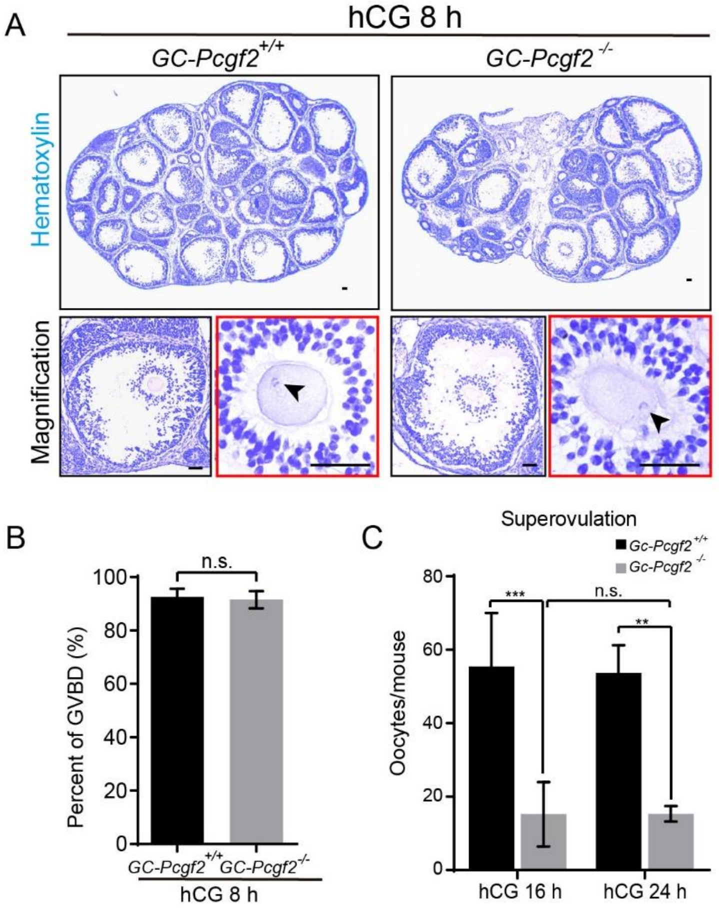

Supplementary Figure S3. ***GC-Pcgf2*$^{-/-}$ mice undergo normal meiotic resumption and cumulus expansion in response to hCG but fail to ovulate.** (**A**) Histological analysis showing normal cumulus expansion and meiotic resumption (arrowheads) in *GC-Pcgf2*$^{-/-}$ preovulatory follicles at hCG 8 h. Scale bars: 50 μm. (**B**) Statistical analysis result showing an identical GVBD percentage of *GC-Pcgf2*$^{-/-}$ preovulatory follicles (92% ± 2%) and controls (93% ± 2%; n = 4 per group). The experiments were repeated at least three times. (C) Statistical analysis of the number of oocytes showing that no more oocytes ovulated at 24 hours after hCG treatment (15 ± 2, n = 3) compared with that at hCG 16 h (15 ± 8, n = 9) in *GC-Pcgf2*$^{-/-}$ female mice. The data represent the results (mean ± SD) of the biological triplicate experiments. n.s. P >0.05, **P <0.01, ***P <0.001 by two-tailed unpaired Student's *t* test.

Supplementary Figure S4.

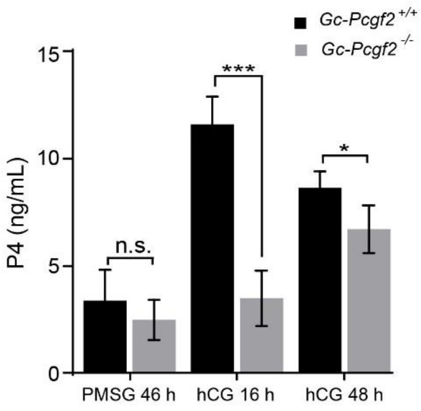

Supplementary Figure S4. Progesterone synthesis is decreased in G*C-Pcgf2*$^{-/-}$ female mice. Hormone detection of progesterone (P4) showing decreased concentrations in *GC-Pcgf2*$^{-/-}$, compared with that in the controls, at hCG 16 h and 48 h (n = 4 per group). The experiments were repeated at least three times. The data represent the results (mean ± SD) of the biological triplicate experiments. n.s. $P > 0.05$, *$P < 0.05$, ***$P < 0.001$ by two-tailed unpaired Student's *t* test.

Supplementary Figure S5.

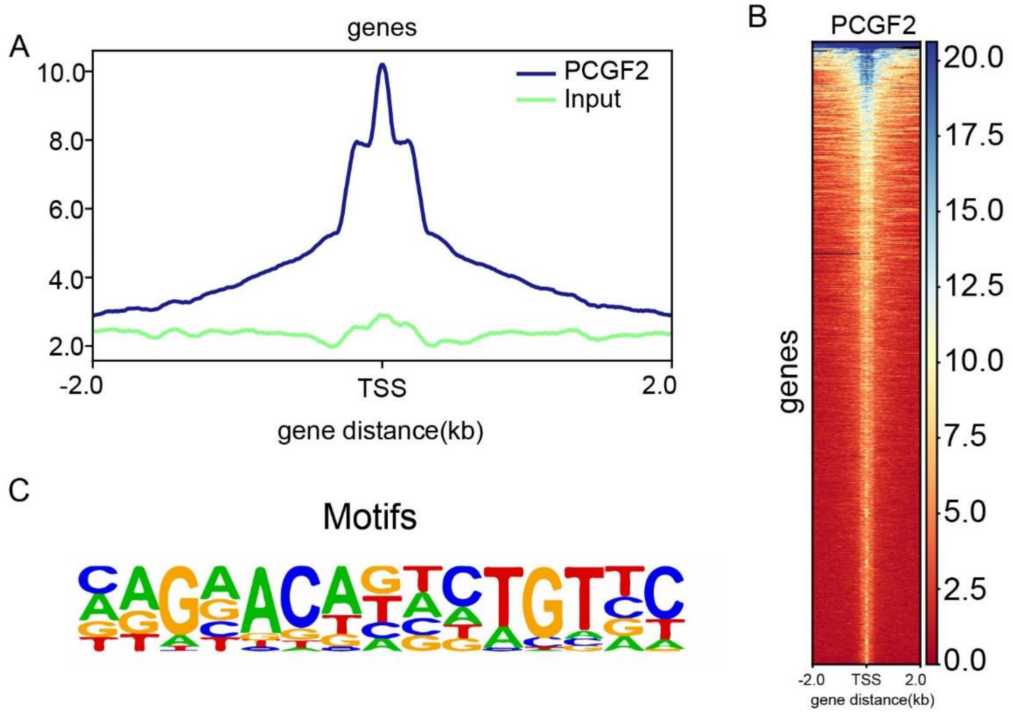

Supplementary Figure S5. ChIP-seq cumulative enrichment deposition centered at peak summit for PCGF2. (**A**) Enrichment profiles of PCGF2 over ± 2.0 kb around the TSS. (**B**) Visualization of PCGF2 enrichment showing the normalized ChIP-seq intensity for PCGF2 proteins over ± 2.0 kb around the TSS. (**C**) Known motif discovery analysis performed underneath the summit of PCGF2 peaks. Sequence weight matrixes of prediction compared with match DNA binding motif is shown. The ChIP-sequence data of PCGF2 was downloaded from Scelfo et al., 2019 (GEO: GSE122715).
